# Design, Development, and Evaluation of an mHealth App for Reporting of Side Effects During Cytostatic Treatment: Usability Test and Interview Study

**DOI:** 10.2196/47374

**Published:** 2023-10-19

**Authors:** Emil Aale Hægermark, Nina Kongshaug, Sunil Xavier Raj, Eva Hofsli, Arild Faxvaag

**Affiliations:** 1 Department of Neuromedicine and Movement Science Faculty of Medicine and Health Sciences Norwegian University of Science and Technology Trondheim Norway; 2 Department of Clinical and Molecular Medicine Faculty of Medicine and Health Scienes Norwegian University of Science and Technology Trondheim Norway; 3 Cancer Clinic St Olavs University Hospital Trondheim Norway

**Keywords:** mobile health, mHealth, user-centered design, usability testing, cancer, side effects, cytostatic treatment, intervention, mobile app, usability, user interface, user, smartphone, mobile phone

## Abstract

**Background:**

Using mobile health (mHealth) interventions such as smartphone apps to deliver health services is an opportunity to engage patients more actively in their own treatment. Usability tests allow for the evaluation of a service by testing it out on the relevant users before implementation in clinical practice.

**Objective:**

The objective of this study was to design, develop, and evaluate the user interface of an app that would aid patients with cancer in reporting a more comprehensive summary of their side effects.

**Methods:**

The usability test was conducted by exposing patients with cancer to a prototype of an mHealth app that allowed for reporting of side effects from a chemotherapy regimen. After solving a set of 13 tasks, the test participants completed a system usability scale questionnaire and were interviewed using a semistructured interview guide. The interviews were later transcribed and analyzed.

**Results:**

The 10 test participants had a mean age of 56.5 (SD 7.11) years. The mean total task completion time for the task-solving session was 240.15 (SD 166.78) seconds. The calculated system usability scale score was 92.5. Most participants solved most of the tasks without any major issues. A minority reported having difficulties using apps on smartphones in general. One patient never achieved a meaningful interaction with our app prototype. Most of those who engaged with the app approved of features that calmed them down, made them more empowered, and put them in control. They preferred to report on side effects in a detailed and concise manner. App features that provided specific advice could provoke both fear and rational action.

**Conclusions:**

The user tests uncovered design flaws that allowed for subsequent refining of an app that has the potential to enhance the safety of patients undergoing home-based chemotherapy. However, a refined version of the app is unlikely to be of value to all patients. Some might not be able to use apps on smartphones in general, or their ability to use apps is impaired because of their disease. This finding should have implications for health care providers’ overall design of their follow-up service as the service must allow for all the patients to receive safe treatment whether they can use an mHealth app or not.

## Introduction

Mobile health (mHealth), the use of mobile devices to support the practice of medicine and public health [1], is currently being explored as a means to promote individuals’ health and well-being, obtain health-related information from individuals and populations, develop and apply medical technology, enroll, retain, and make patients adhere to a care program, and create a digitalized health care arena through which patients can communicate with health care professionals [2].

mHealth is a promising avenue of research and development that has caught the attention of health policy makers [3]. World Health Organization now recommends “digital tracking of clients” health status and services (digital tracking) combined with decision support in “settings where the health system can support the implementation of these intervention components in an integrated manner; and for tasks that are already defined as within the scope of practice for the health worker.”

In 2017, tackling medication-related harm was announced as a global patient safety challenge [4]. World Health Organization highlighted 3 priority areas: high-risk situations, polypharmacy, and care transitions. High-risk situations point to medications that are particularly liable to produce adverse events. They tend to have a narrow therapeutic index, meaning that minor dosing errors can cause catastrophic outcomes [5]. Chemotherapeutic agents belong to this group [6].

A cancer clinic that prescribes a cytostatic drug that is supposed to be taken in an outpatient setting needs to be arranged so that the patient can take the drug with a minimum of risks. In the event of potentially harmful side effects, risk must be evaluated, communicated, and mitigated. To carry out the risk-mitigating activities, like showing up at the hospital or postponing taking the drug, the risk must be evaluated and communicated to the patient in an effective manner. mHealth apps could be the solution to these problems [7]; however, their design needs to be appropriate.

To achieve the most likely scenario for successfully implementing mHealth interventions into clinical care, they should be evaluated during the development stage [8]. User-centered design of mHealth apps can be described as a design process that revolves around creating a design based on the abilities of the patient (the user), not the clinician or the app developer [9]. The involvement of the potential users, through usability tests, minimizes the risk of creating an unusable app [10].

A key feature of an app to enhance the safety of taking a cytostatic drug is the app’s user interface for reporting side effects. In an earlier study of such an app, we found that a user interface that provided a simple yes or no option led to an underreporting of side effects [11]. In the interviews, patients revealed that they refrained from reporting side effects out of fear of having to stop taking a drug that they saw as a potentially life-saving therapy.

The objective of this study was to design, develop, and evaluate the user interface of an app that would aid patients in reporting a more comprehensive summary of their side effects.

## Methods

### Study Design

The study was designed as a usability evaluation with patients with cancer as test users and informants [12]. Each test was conducted individually. Participants tested a prototype of an mHealth app and were instructed to simulate the reporting of side effects from chemotherapy treatment. After completing the task-solving part, the participants completed a system usability scale (SUS) questionnaire [13] and conducted a semistructured interview based on their experiences with using the prototype app. Since the objective of the study was to explore the user interface of the prototype, a formative assessment process was used [14].

### Ethical Considerations

The Regional Committee for Medical and Health Research Ethics Central Norway confirmed that their approval was not required for this type of study (REK 2020/173492). The Norwegian Center for Research Data approved the study in March 2021. Participants provided written and oral informed consent before participating. Any sensitive data were kept in a storage room with restricted access. Records were transcribed, and the transcripts were anonymized. The records will be deleted after publishing the results. Participants received no compensation except reimbursement of travel expenses.

### Inclusion and Exclusion Criteria

The inclusion criteria were: at least 18 years old, in an outpatient setting at the cancer clinic, received or have recently received capecitabine, and had a smartphone or a similar device (like a tablet) that he or she used regularly.

Patients were excluded if one of the following situations were present: not experienced any side effects when treated with capecitabine, already participated in another usability study with the app and therefore familiar with the tasks, or lacked fundamental Norwegian language skills.

### Recruitment Procedure

Patients were recruited from the outpatient department of the Cancer Clinic at St. Olavs Hospital in Trondheim, Norway. The physicians presented the project to the eligible patients when they visited the hospital for a consultation. Interested participants met with one of the research members who provided a more detailed presentation. Afterward, the patients who wanted to participate gave written informed consent, and a suitable date and time for the test were arranged. The recruitment stopped when saturation was reached [15].

### The Prototype App

**Figure 1:**
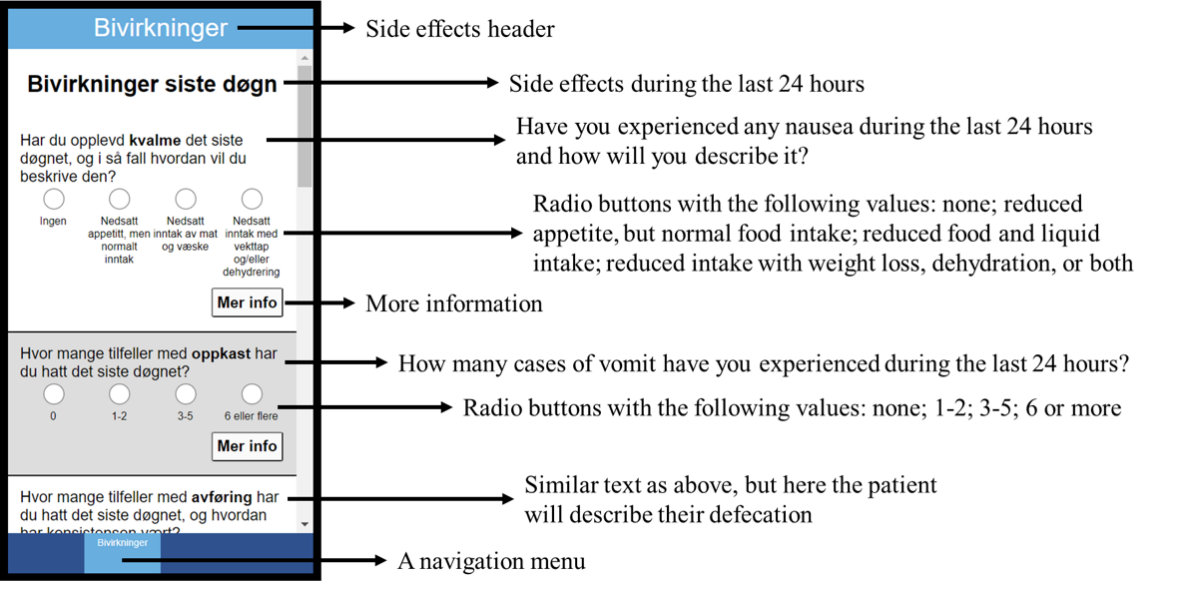
The radio buttons interface when selecting different values for each side effect.

**Figure 2:**
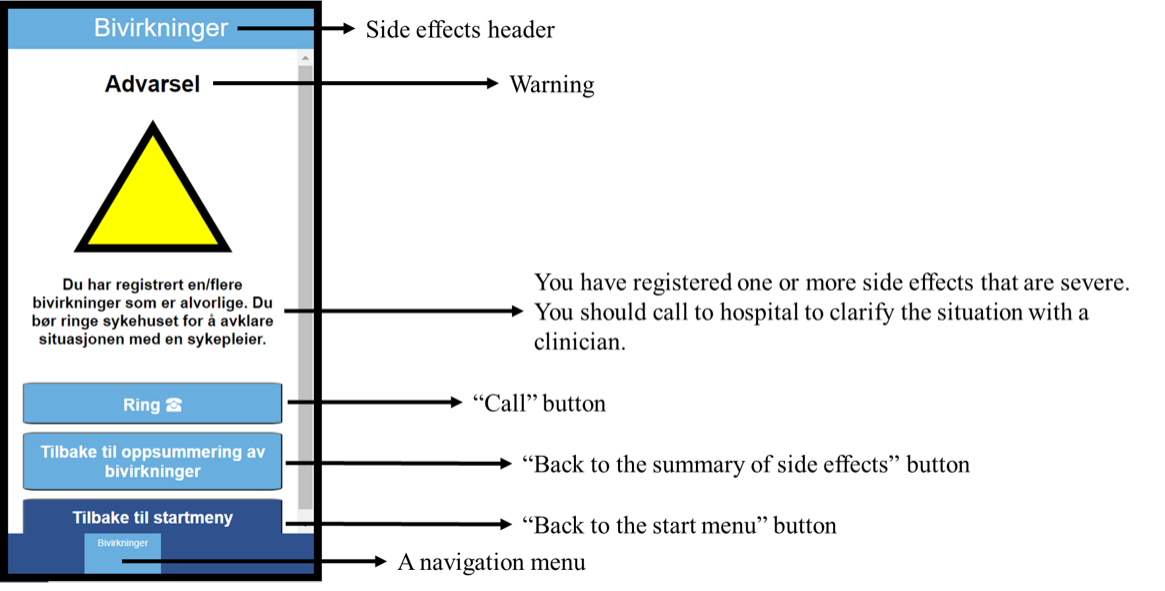
The warning with a yellow triangle when reporting at least one severe side effect.

The prototype app was created as a web-based app using HTML, CSS, and JavaScript, using the already existing app as a foundation [11]. It was designed to mimic a regular smartphone page (Figures 1 and 2). After each test session, if necessary, changes were made to the app. The research group provided smartphones for the usability test sessions.

### Task Development and Task-Solving

A preliminary list of tasks was developed by going through the functions that were present in the previous version of the app [11] and using existing literature [16-18]. Thereafter, physicians and patients tested out these preliminary tasks and provided feedback. The resulting 13 tasks are described as part of Textbox 1. The tasks varied in complexity, and they were supposed to solve the tasks by themselves and not ask for help. The participants were observed by one of the research members (EAH) during the task-solving session. The observer’s role was to note how each task was interpreted and solved. The participants were encouraged to think aloud when solving the tasks.

**The tasks.**

**Task 1**: Report a set of side effects

**Task 2**: Register a set of values for each side effect

**Task 3**: Find more information about a specific side effect

**Task 4**: Send in the set of side effects

**Task 5**: Check formerly reported side effects

**Task 6**: Check the side effects reported for this day and go back to the main menu

**Task 7**: Report a set of side effects for a day where a report has already been done

**Task 8**: Register a set of values for each side effect

**Task 9**: Change a specific side effect

**Task 10**: Go to the summary page

**Task 11**: At the summary page, change a specific side effect

**Task 12**: Send in the set of side effects

**Task 13**: Call the hospital

During the task-solving session, participants were presented with different design options for the interface. When reporting side effects, they were presented with 3 different interfaces: radio buttons (Figure 2), yes and no buttons (based on questions that differentiated between grade 2 and grade 3 on the Common Terminology Criteria for Adverse Events) [19], and drop-down menus. Another design option was the interface of the calendar. The third design option was the design of the warning when reporting a severe side effect and the need to call the hospital. It was presented with a text either with a yellow triangle (Figure 2) or not. All these different design options are presented in Multimedia Appendix 1.

### System Usability Scale

After completing the tasks for the prototype, participants filled in a SUS questionnaire created by Brooke [13]. Help was provided if they needed help interpreting the different assertions in the questionnaire.

### The Semistructured Interviews

The last part of the test session consisted of a semistructured interview. The interviews were conducted by EAH. An interview guide was used as the basis for the interviews, presented in Multimedia Appendix 2. The topics primarily focused on the perception of the prototype, the design options, and the navigational considerations. These topics were inspired by interview guides from other usability studies [17,18] and literature on qualitative studies [9,15]. The interview guide was preliminarily evaluated on other patients and physicians who provided feedback. The interviews were conducted in Norwegian.

### Recording and Transcribing

The test sessions were recorded on both audio and video. They were placed on each side of the participant. The video recorder was placed in such a position that it could only record the screen of the smartphone. Both recordings were used when writing the transcript for each test session. EAH created the transcripts. The transcripts were written in Norwegian.

### Data Analysis

The task-solution part sampled 2 main parameters, task completion time and the degree of completion [14]. Task completion time was further divided into total task completion time and time used for each task. The time used for each task was measured as the interval between the time the interviewer had finished reading it and the time the user had completed it. The time use parameters were presented as means and SDs (using R; R Foundation for Statistical Computing).

The degree of task completion was graded based on 3 outcomes [14]: task completely solved without any help or navigation mistakes, task solved with navigation mistakes but without any help, and task not solved or solved with help. The division between the last 2 types of mistakes was to differentiate between critical and noncritical user errors. The number of participants who ended up with each outcome was used to measure the degree of completion (using R). The SUS score was calculated using the standardized formula [13].

The transcripts were analyzed by both EAH and AF (the supervisor). Data were analyzed according to systematic text condensation [20]. EAH and AF read through them and identified initial codes that were collated to create the concepts individually. These concepts were later discussed and compared, and together the main themes were constructed. NVivo (version 1.6.1; Lumivero) was used to structure the code and concepts. The codes and concepts were identified preliminarily in Norwegian before being translated into English. Relevant quotes in this paper were translated and presented in the results.

## Results

The overarching findings from the usability tests were (1) some patients could not learn how to interact with the app, (2) those who managed to engage with the app preferred to report on side effects in a detailed and concise manner, and (3) the way the app thereafter sought to advise or instruct the patient could provoke both rational and irrational behaviors.

### Participant Demographics

The study recruited 10 participants between August and November 2021 (Table 1). The mean age was 56.5 (SD 7.11) years. In total, 7 of the participants were females. Half of the participants had “high school” as the highest completed education level, while the other half had completed university or college. No participants had just completed elementary school. Regarding hours per day with a smartphone or computer, 6 participants stated that they used 1 for more than 3 hours, 2 participants answered between 1 and 3 hours, and the last 2 said that they used 1 for less than 1 hour.

**Table 1:** Participant demographics.

		Values
Age (years), mean (SD)		56.5 (7.11)
Female, n (%)		7 (70)
**Highest education completed, n (%)**		
	High school	5 (50)
	University or college	5 (50)
**With computer or cellphone (hours per day), n (%)**		
	0-1	2 (20)
	1-3	2 (20)
	3+	6 (60)

### Assessment of Task Completion Time, Degree of Completion, and SUS Score

**Figure 3:**
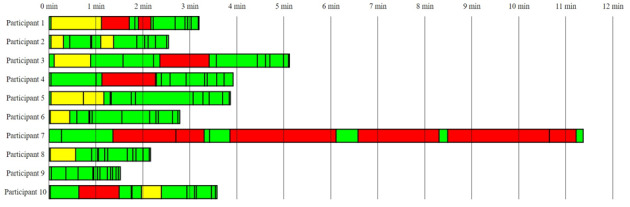
Task completion time and degree of completion. Green: solved completely; yellow: solved with navigation mistakes; and red: not solved or solved with assistance.

Task completion time and degree of completion are presented in Table 2. Figure 3 visualizes time use and degree of completion per test participant. The mean total time use was 240.15 (SD 166.78; range 91.0-682.0) seconds. The calculated SUS score was 92.5. The mean SUS scores for each item are presented in Multimedia Appendix 3.

**Table 2:** Task completion time and degree of completion.

Task number	Values (seconds), mean (SD)	Degree of completion, n (%)		
		Solved completely	Solved with navigation mistakes	Not solved or solved with help
Task 1: Report a set of side effects	4.0 (4.46)	10 (100)	0 (0)	0 (0)
Task 2: Register a set of values for each side effect	40.4 (18.05)	4 (40)	6 (60)	0 (0)
Task 3: Find more information about a specific side effect	29.55 (23.38)	6 (60)	1 (10)	3 (30)
Task 4: Send in the set of side effects	24.25 (19.27)	8 (80)	0 (0)	2 (20)
Task 5: Check formerly reported side effects	2.75 (2.73)	10 (100)	0 (0)	0 (0)
Task 6: Check the side effects reported for this day and go back to the main menu	17.65 (17.8)	8 (80)	0 (0)	2 (20)
Task 7: Report a set of side effects for a day where a report has already been done	25.1 (40.4)	7 (70)	2 (20)	1 (10)
Task 8: Register a set of values for each side effect	33.45 (17.83)	10 (100)	0 (0)	0 (0)
Task 9: Change a specific side effect	20.1 (29.75)	9 (90)	0 (0)	1 (10)
Task 10: Go to the summary page	4.8 (2.73)	10 (100)	0 (0)	0 (0)
Task 11: At the summary page, change a specific side effect	24.15 (37.6)	9 (90)	0 (0)	1 (10)
Task 12: Send in the set of side effects	10.4 (8.8)	9 (90)	0 (0)	1 (10)
Task 13: Call the hospital	3.55 (3.62)	10 (100)	0 (0)	0 (0)

### Assessment of Task Performance

As can be seen from Table 2, 7 out of the 10 participants made 2 or fewer mistakes. Test participants struggled most with tasks 2, 3, and 7. Only 4 accomplished solving task 2 (register a set of values for each side effect) without assistance from the test conductor. In total, 6 of 10 users used yes-no buttons at their first iteration when they were asked to grade the severity of each side effect. The mistakes were due to selecting the wrong value based on the story and therefore classified as navigation errors. Task 3 (find more information about a specific side effect) tested whether the users were able to locate a “More information” button located at the bottom right of the interface (Figure 1). Three of the users failed to locate the button at all and ended up returning to the start menu and needing assistance. One user pressed the wrong “More information” button. Three users struggled with task 7 (revise an existing side effect report) as they did not comprehend that the “Report side effect” button also could be used to modify a side effect report that already had been created. The 2 with navigation mistakes ended up exploring the calendar looking for a way to overwrite before they returned to the main menu and clicked the correct button. The one who needed assistance seemed unable to comprehend the question.

Taken together, the user tests revealed rather minor issues with the user interfaces. The patients approved of many features of the app and how they could make use of them to improve their situation as patients with cancer on chemotherapy. This is further elaborated in the following sections.

### The Ability to Learn How to Use the App

The usability tests quickly revealed that some patients struggled with carrying out the tasks using the app. One of the patients spoke about difficulties in using apps on a smartphone in general:

Some people are great at downloading apps for their smartphones, while others are not. I am not good at downloading apps. Consequently, I am not able to download such apps. Recently, I was supposed to download an app for my fitness gym. So, I went to the gym and asked a young person to help me and explain everything. Otherwise, I would go to my son who helps me.

Patient 4

In this case, the inability to learn how to use the app was rooted in factors within the patient. Another patient reported having dyslexia, which influenced his ability to interact with the app. A third reported that the cancer therapy had reduced her mental capacity. She felt that her “brain was slowing down,” and that this impaired her performance during the tests. When observing these patients, they particularly struggled with tasks that required navigation through multiple elements, like tasks 2 and 6, when they were exposed to the user interface for the first time. These navigation mistakes were more or less absent during the second part of the test session. However, this group spent more time on these tasks compared to the other participants.

Of those capable of using the app, all stated that it was, in general, easy to use. As the test was divided into 2 parts, some tasks from the first part were repeated in the second. It was observed that the test patients were much less likely to make navigational errors in the second part. Hence, they quickly learned how to engage with the app. Some user interfaces were not used as intended during design. For instance, when trying out the functionality for reporting drug-related side effects, pushing the “Back to the main menu” button caused the app to go back to start without saving the reported side effects, leading to resetting the values for the side effects, and subsequently, the need for the participants to fill out each value a second time. One participant suggested the following improvement:

I think so [that a warning should appear]. Then I would have known that I had made a small mistake and that I needed to adjust.

Patient 7

Another issue regarding the user interface had to do with the text that was attached to certain buttons. The most prominent one was the buttons that allowed the user to re-enter a value of a side effect. These buttons had the text “Reset” (Norwegian: “Nullstill”) written inside of them. Consequently, many participants became unsure whether pressing these buttons allowed them to change the specific element or if pressing them would reset the whole page. In addition, here, they had suggestions for improvements:

I believe that “Change” [instead of “Reset”] makes more sense. Then you know what that button does, and it is easy to understand. Instead of a “Reset”-button, which might make people believe that the whole page would reset.

Patient 8

### Patients Preferred to Report on Side Effects in a Detailed Manner

A majority of the test patients preferred the user interface alternative that allowed them to provide a graded description of their side effects:

I believe that it often happens that you get a question that could be difficult to answer with a “Yes” or a “No”. In addition, the world is not “yes” or “no”. Consequently, I believe it is better to be provided with alternatives.

Patient 4

This was accomplished by adding radio buttons that presented a Likert-type scale for grading the side effects (Figure 1). Another element that patients highlighted as important was a page that enabled them to review and control what they were about to report to the hospital:

With all these technological interventions, there is uncertainty about whether everything has been done correctly or not. In addition, it [the summary section that summarizes the report] is easily structured and quick to read through. It is reassuring.

Patient 3

Regarding the option to press a button to receive information about each side effect, participants approved such functionality, as it enabled them to learn more about the medication they were taking, being another factor that could contribute to their safety. One of the participants expressed that they would like to see more statistics for each side effect, like prevalence among similar patients. On the other hand, some users were concerned by the amount of information on each site. One participant described it as “information overload,” making him or her frustrated and tired because he or she was unable to comprehend the whole page.

One participant was concerned with using such apps during chemotherapy treatment as they expressed a need for human contact during this challenging time of their life. They feared that an intervention like this would replace how they got into contact with health care professionals today and that their treatment would be more automatic and robotic. One participant stated the following:

I am very satisfied with the treatment I have received. I have learned that humans are the most important. The calls I have made with the hospital and the feeling that I have been able to ask questions regarding my side effects made me feel like I was not just another number, but I have felt like me. I experienced that the nurses were very concerned regarding the human being.

Patient 10

### Being Alerted to Take Action

The most controversial design was that of a warning that was presented to the user when they were about to report particularly severe side effects. Some patients stated that the warning was appropriate because it would make them understand more fully the severity of the situation:

You think that this is a serious situation and that you need to make a call. Often one might need this message because one might think that the situation might not be as dangerous as one considers. That is what I thought, which made me not make a call. But with this warning (with the triangle), it confirms that I must call the hospital.

Patient 2

Hence, appropriate warnings could prevent patients from downplaying the situation. Other patients reacted with anxiety:

You become scared and start to think: Am I going to collapse this very minute? When you are as sick as you are, and you get a warning that says that you are not well, I believe it is important to be a lot more psychological with the message, being more kind and friendly.

Patient 3

Among those who reacted with anxiety, some preferred a less strict warning without the yellow triangle and with a more helpful and hopeful text. Others stated that an app that provoked anxiety could lead them to abolish both the treatment and the app:

I do not think that you should use warnings unless it is necessary, because we have been given the confidence that we can receive this treatment from our own homes. You should not use this fear, we do not need that. If you get such a warning, some patients might think: “This is terrible, I do not want to do this.” And you become anxious instead.

Patient 3

Furthermore, participants would prefer to not be presented with details on the dangers of a particular side effect situation. Instead, they preferred to be told what to do:

I feel like I do not need to know what stands there. When it says severity 3/3, I would have thought, “Oh, am I going to die now?!.” I believe it is unnecessary to have it because when you submit the side effects report, a message suggesting that you should call will appear if required.

Patient 6

## Discussion

### Principal Findings

In this study, we have found that conducting user tests with real patients can be used to uncover design flaws and subsequently refine and adapt an mHealth app that intends to enhance the safety of patients who undergo home-based treatment with a cytostatic drug. We have found that patients approved of features that calmed them down, made them more empowered, and put them in control. An improved version of the app will likely assist patients in creating and relaying more comprehensive, complete, and reliable side-effect reports and help them take more rational action. Somewhat paradoxically, app features that provided specific instructions or advice could provoke anxiety. A third category of insights relates to the population from which our very small sample was drawn: some patients were not very capable of using apps on a smartphone in general or stated that their disease (cancer) had a negative impact on their ability to learn how to use an app in a meaningful way. This will have implications for the overall design of the service.

### Patients Approved of Features That Calmed Them Down, Made Them More Empowered, and Put Them in Control

The usability tests revealed that the patients preferred designs that provided them with control, which allowed for more nuanced reporting of side effects and the ability to review and revise what they were about to report to the hospital. Both can potentially contribute to patient empowerment as they provide the patient with the knowledge that is required to make rational actions [21]. By using radio buttons, patients were allowed to report a more nuanced and hence realistic picture of their situation. This design choice is found in many other mHealth solutions [22-24] and contributes toward a comprehensive and more accurate picture of patients’ side effect experiences. The summary section provided a calming effect as well because this feature aided the patient in achieving an overview of their situation and what actions could be taken. Additionally, having small messages appear when the patients misclick, that is, going back to the start menu and resetting their reported values, could act as insurance and help guide them through the app. Another option could be to temporarily save an incomplete report when they go back to the start menu and later let them continue the report. We believe that an awareness of such features in the design of the user interface is a necessary step in order to maximize user satisfaction and usefulness.

These findings emphasize the benefit of having future users (of the service) assess candidate user interface designs as early as possible. Having a study population that resembles the future patient population of users will secure transferable results [15]. This approach is consistent with that of many other developers: in an effort to create an app that caters to the needs of the target users, usability testing should take place before offering the app to a larger group of patients [17,22,25-27].

### The Design of Features That May Provoke Anxiety

The value of an app that intends to enhance medication-related safety hinges on its ability to achieve a rational reporting of side effects, aiding the patient to make decisions and take rational action. App user interfaces that subsequently communicate medication-related risks must be designed so that the patient thereafter takes rational action. As this study shows, despite few participants, the population was quite heterogeneous in terms of the preferred warning. The same message could trigger fear among one group, while it could lead to trivialization in the other. Both of these extremes could lead to misuse of the app and cause potentially lethal events. We see no quick design fix to accommodate this heterogeneity. A potential solution lies in a redesign of the whole service: A solution that aids the patients in reporting the side effects but where it is the responsibility of the hospital to react to the report could provoke less fear. Another option could be to let the patients themselves customize the app at the start of the treatment with the help of a health care provider. This self-customization could be based on a set of questions about technical proficiency, personality of the patient, passive or active preferences, etc. However, these questions will require iterative development in order to secure the most accurate data and optimal app use for each user. With the use of more personalized health care regarding medication for certain cancers, mHealth apps could be an avenue where customization could be useful as well.

Patients with the same disease having different preferences when it comes to the design of features in an mHealth app have been reported by many others [11,28-31]. To our knowledge, we are the first to report that seemingly small differences in patient needs and preferences can have profound impacts on the value of an app whose main feature is the communication of medication-related risks. In our opinion, the potential for a safety-critical feature to provoke irrational behavior has implications for the design of mHealth evaluation studies.

### Factors Outside the App Will Preclude Some Patients From Benefiting From the Service

While this study only contained 10 participants, it showed that the target users differed highly concerning the ability to use apps on a smartphone. While most of the participants were able to use the prototype with almost no issues and were able to complete the task quickly, some of the users struggled and spent a longer time completing the tasks. For instance, the quickest participant spent 91 seconds, while the slowest spent 682 seconds. The degree of completion varied greatly as well, 7 participants solved the tasks with 2 or fewer mistakes. On the other side, one of the participants made 6 mistakes.

This implies that the mHealth app is unlikely to be used by all patients in the target group. Factors that affect their performance can be divided into 2 groups. One group consists of factors that were present before the disease, and the other consists of factors that are affected by the disease. Among the first group, factors that caused more mistakes were, for instance, less experience in using apps on a smartphone, or other handicaps, like dyslexia. In the other group, one factor is the reduced mental capacity caused by the disease, which was also found in Das et al [32] study. Furthermore, such obstructive factors might affect the patients’ commitment to using the app, because they feel that they cannot become proficient with the app. To avoid some of these mistakes, patients will be receiving education and information about the app before use.

In addition, this indicates that “one size does not fit all,” and it is consistent with other studies, where former experience with smart devices to a great extent affects their performance compared to the less experienced [16,17,24,25,33]. For instance, Aiyegbusi et al [17] had similar results when they tested out an electronic patient-reported outcome measurement system for patients with kidney disease, whereas Hanghøj et al [24] conducted a usability test with adolescents and young adults with cancer. Easy and understandable elements and navigation were critical for the user experience. For the group of participants that struggled with the app, the struggle seemed to be rooted partly in comprehending the user interface. Consequently, trying to refine the user interface to accompany their needs (ie, their reduced mental capacity) increases the chances of a successful implementation.

### Implications for the Overall Design of the Service

**Figure 4:**
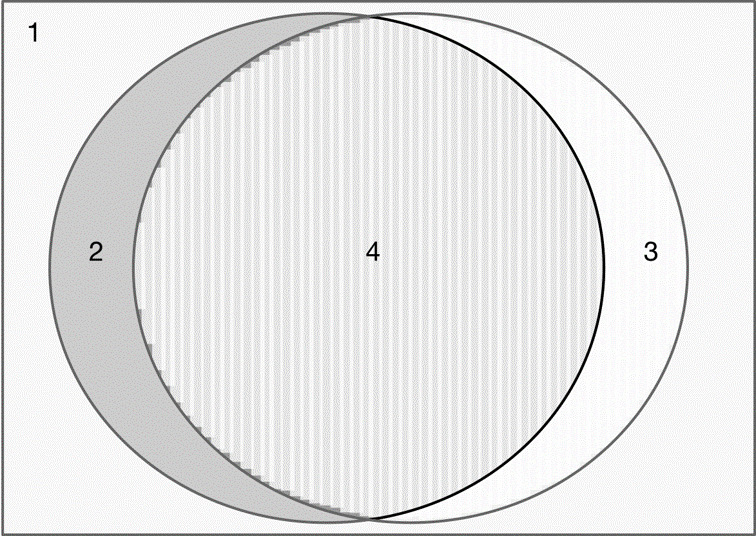
Venn diagram of the population of patients with cancer. (1) The whole population of patients with cancer, (2) the patient population that wants to use the app, (3) the patient population that has the skills that are required to use the app, and (4) the patient population that is capable of and actually wants to use the app.

In our opinion, this has implications for the overall design of the service. We see that the target population is highly heterogeneous and that the context of the patient’s use of the app has an overwhelming influence on the user experience. We have learned that patients are more or less skilled in using apps on smartphones and that they differ in learning abilities and motivation. This mHealth app stands in stark contrast to other health-related apps, for instance, a fitness app [34]. Individuals using a fitness app are relatively healthy and not in a situation where a disease threatens to shorten their life. These mHealth interventions that are meant to support the treatment of a serious disease have a completely different context. We believe that this will contribute to the patient’s motivation to use the app, as they are in a life-threatening situation and are asked to use the app by the health institution that provides the care that can save their lives. In other words, the motivation for using the app could be shaped by their health status and the necessity of taking a drug that might cause harm. Within the cancer population, there will always be users who prefer to have a nurse calling them regularly through a treatment period. With the lack of enough personnel in health care, it will not be feasible to have such a service for the whole group because having nurses calling patients regularly demands considerable resources. Figure 4 demonstrates how the population of patients with cancer can be divided regarding whether they can use an mHealth app in their treatment.

**Figure 5:**
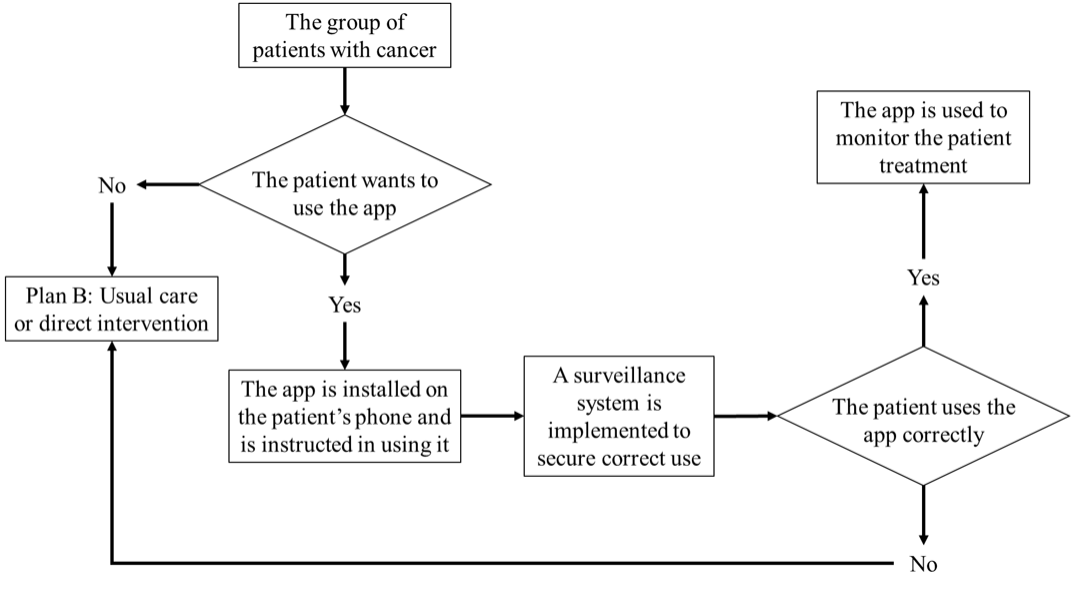
Proposed plan for the service or workflow. The group of patients with cancer is offered to use the app. Those who chose to use it will be educated on using it. The additional surveillance system will monitor that the patients use the app correctly. Those who refuse to use the app or use it incorrectly will receive the usual care instead.

To create a safe environment for patients treated with chemotherapy, we therefore now believe that the app itself is not enough. There will be users who will fail to use the app correctly either due to trepidation or erroneous use of the app. In addition, we believe that a surveillance system should be implemented that tracks and ensures the correct use of the app. For those patients who use the app correctly, the app will be the primary treatment-supporting tool. On the other hand, for those patients who fail to use the app correctly, like forgetting to report side effects, the relevant health service needs to be alerted and move the patient from using the app to another treatment option, for instance, the direct intervention approach. A map of the workflow with the app included in such systems is outlined in Figure 5.

### Further Work on the App and Preliminary Designs of Forthcoming Evaluation Studies

The results of this study will be applied in the development of a new version of the mHealth app, together with building the surveillance system, which we will deploy to a small number of patients for a new test in their home environment. Such an experiment will resemble a phase 1 clinical trial [35]. The trial should explore the effect of the redesigned app on the correctness and severity of the side effect reports, on the comprehension of the patients of his or her peculiar situation, and whether the use of the app made the patients feel that they were taken care of by the clinic. Hopefully, using the refined app would improve patient outcomes (like fewer unplanned hospital admissions), quality of life, and communication with health care providers, as the side effects reporting would be on a daily basis that both the patients and the health care providers could track and react upon. This experiment should be carried out upon the completion of a redesign of the entire service.

In the redesign of the app, we wish to explore a more population-based approach [36]. By this, we want to create a new division of labor between the patient and the clinic that is responsible for the entire service. In a population health approach, data collection is organized so that the clinic can take into account the health status of the entire group of patients and react toward those patients who need to be reacted upon [37]. In a service that is built around the voluntary use of an app, both patients’ use and nonuse of the app could be monitored and used to adapt and personalize the service.

### Limitations

Usability studies do have some inherited weaknesses [9]. First, the tests themselves happen in an artificial setting and not in the clinical environment. To handle this the best way possible, we tried to create as realistic tasks as possible. Additionally, it is unsuitable to include the whole patient population when conducting a usability study. Nevertheless, we believe that our inclusion and exclusion criteria have made it possible to get a fairly representative population. Moreover, the participants in usability testing might be more familiar with and interested in such interventions. Consequently, they score higher than the average human. While conducting usability testing may take some more time, in the end, the finished product is more likely to resonate with the expectations of the patient and increase user satisfaction [8].

### Conclusions

We conclude that it is possible to use a user-centered approach to create an app where patients with cancer can report their side effects. We discovered that the group of patients with cancer should be divided into 2 primary groups, where the first group uses the app efficiently, while the other group cannot. Consequently, these 2 groups required different follow-ups from their health care provider. Regarding the design of the app, the app should aim to report side effects in a detailed manner. Even if patients would prefer to have a nurse calling them regularly through a treatment period, the lack of people in health care will not allow continuing this tradition. Regarding the way the app facilitates communication between the patients and the hospital, further research is needed to secure behavior that leads to reporting of side effects in a rational manner.

## Appendix

Multimedia Appendix 1 The design options.

Multimedia Appendix 2 The interview guide.

Multimedia Appendix 3 System usability scale summary.

## Abbreviations

mHealth: mobile health
SUS: system usability scale

## Data Availability

The data sets generated during and/or analyzed during this study contain personal health information and can therefore not be made publicly available. Anonymized versions of the data material can be provided by the corresponding author upon reasonable request.
