# Supplementary material for: Design, Development, and Evaluation of an mHealth App for Reporting of Side Effects During Cytostatic Treatment: Usability Test and Interview Study
Source: JMIR Form Res. 2023 Oct 19;7:e47374. doi: 10.2196/47374 (PMC10623228; doi:10.2196/47374)
Supplement: Multimedia Appendix 1 [file formative_v7i1e47374_app1.docx]

# **Multimedia Appendix 1.** The design options.

## Reporting the Side Effects


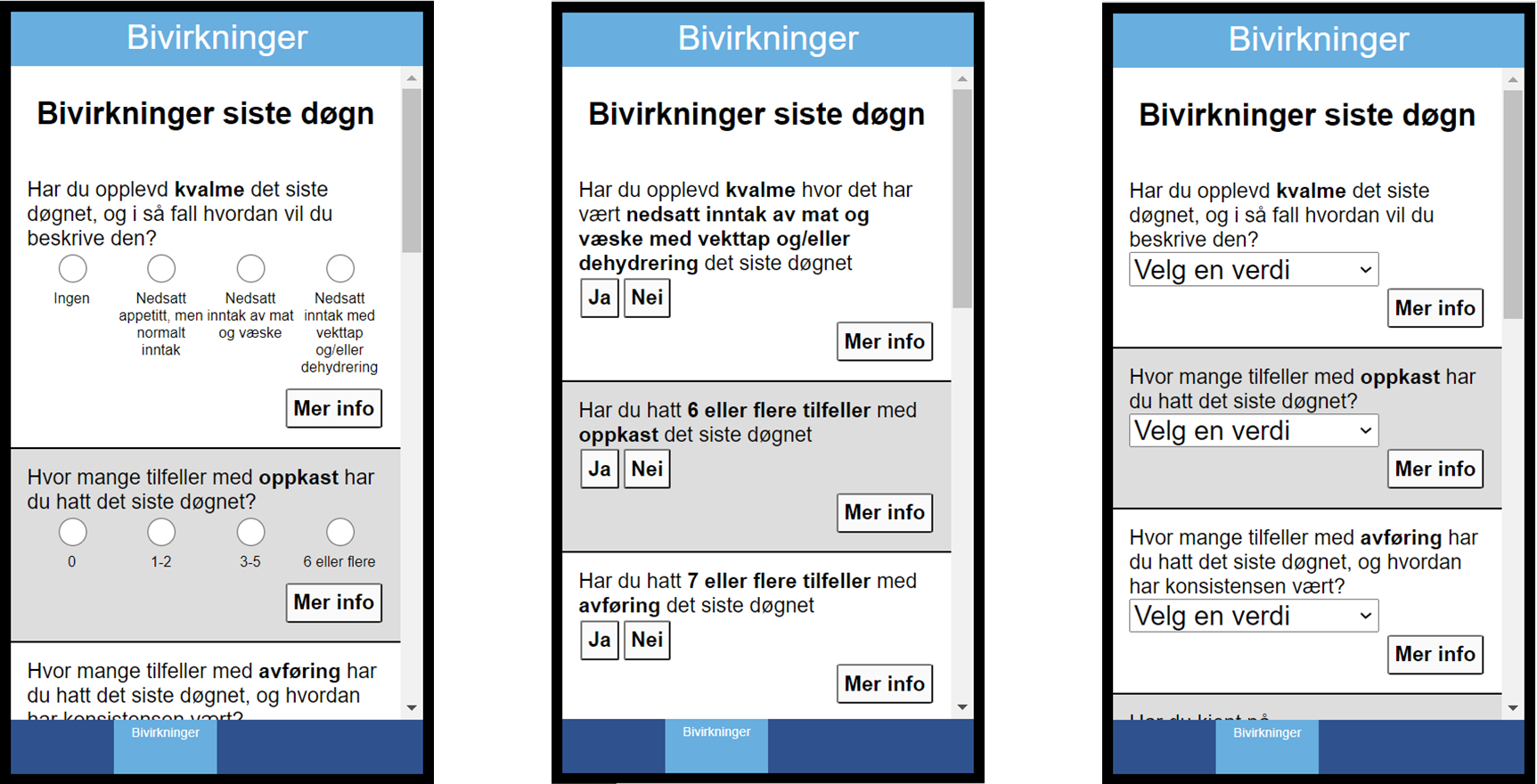


The design options for selecting values when reporting side effects.

From left: Radio buttons, “Yes” and “No”-buttons, & Drop-down menu

## The Calendar


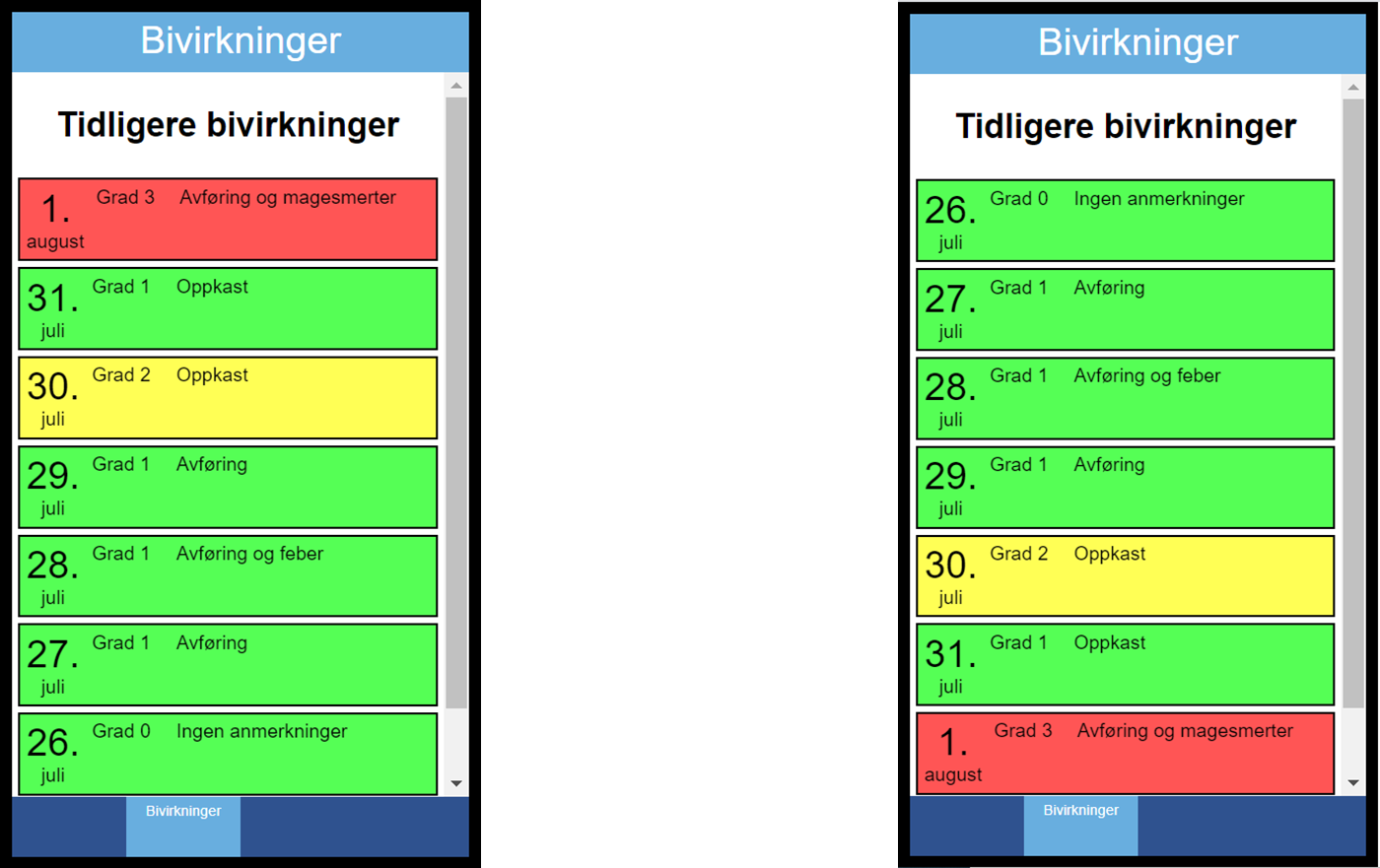


The design options when selecting the order of the report dates in the calendar.

From left: Most recent report on the top & oldest report at the top

## The Warning


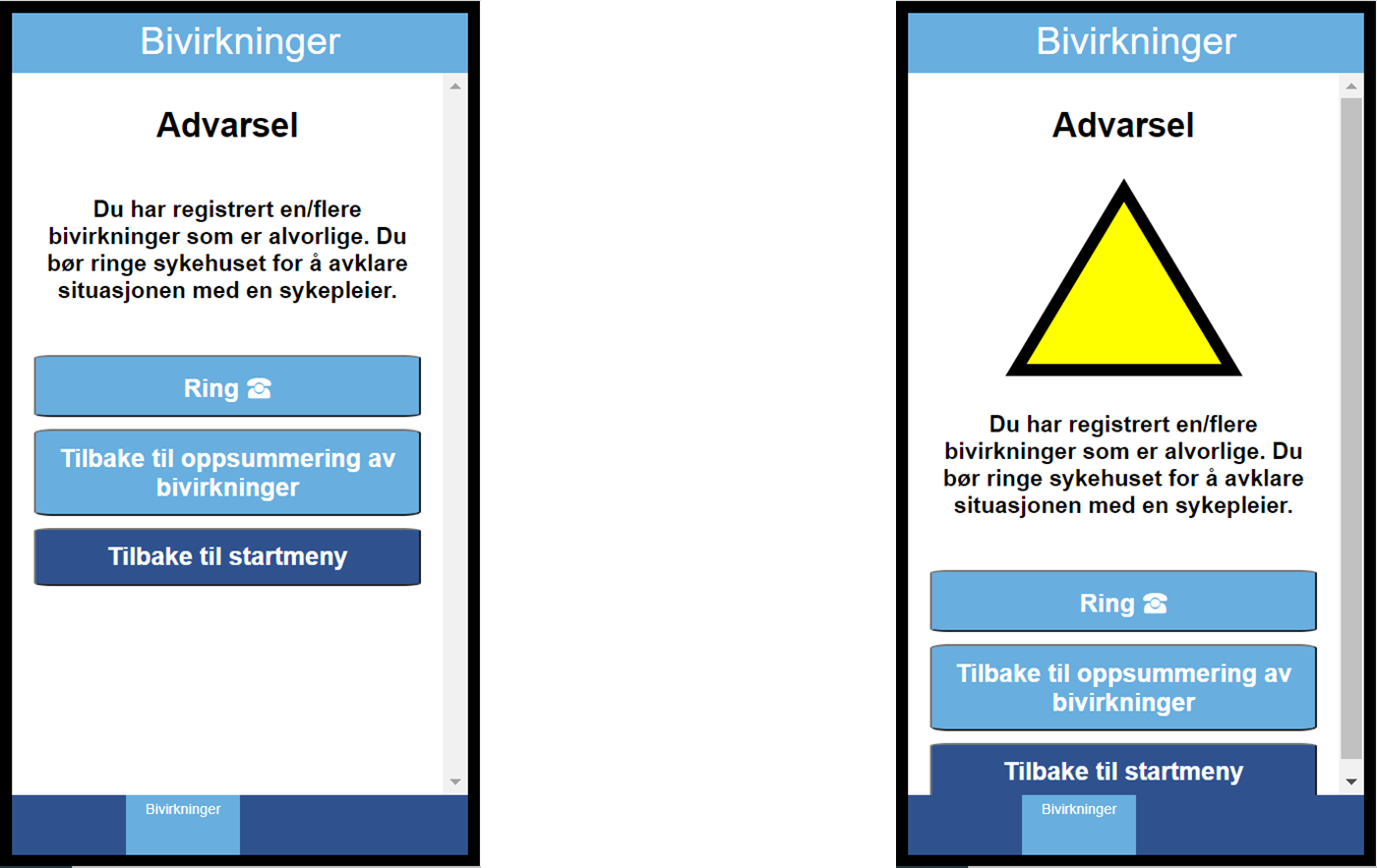


The design of options of the warning when reporting “severe” side effects.

From left: Without the triangle & with the triangle
