# Supplementary material for: Design, Development, and Evaluation of an mHealth App for Reporting of Side Effects During Cytostatic Treatment: Usability Test and Interview Study
Source: JMIR Form Res. 2023 Oct 19;7:e47374. doi: 10.2196/47374 (PMC10623228; doi:10.2196/47374)
Supplement: Multimedia Appendix 2 [file formative_v7i1e47374_app2.docx]

# **Multimedia Appendix 2.** The interview guide.

What do you believe is a suitable time?

Was some takes more difficult than others, and why?

Preferred design options

- Reporting the side effects
- The calendar
- The warning

Display of elements

- Text size
- Size of buttons
- Suitable colors
- Understandable language

Navigational considerations

- Name of buttons
- The summary page
- The text when entering the “More Information”-button
- Errors
