# Supplementary material for: Design, Development, and Evaluation of an mHealth App for Reporting of Side Effects During Cytostatic Treatment: Usability Test and Interview Study
Source: JMIR Form Res. 2023 Oct 19;7:e47374. doi: 10.2196/47374 (PMC10623228; doi:10.2196/47374)
Supplement: Multimedia Appendix 3 [file formative_v7i1e47374_app3.docx]

# **Multimedia Appendix 3.** System usability scale summary.

| Item Name | Mean |
| --- | --- |
| 1. I think that I would like to use this system frequently | 3.7 |
| 2. I found the system unnecessarily complex | 3.8 |
| 3. I thought the system was easy to use. | 3.8 |
| 4. I think that I would need the support of a technical person to be able to use this system. | 3.5 |
| 5. I found the various functions in this system were well integrated. | 4.0 |
| 6. I thought there was too much inconsistency in this system. | 3.6 |
| 7. I would imagine that most people would learn to use this system very quickly. | 3.6 |
| 8. I found the system very cumbersome to use. | 4.0 |
| 9. I felt very confident using the system. | 3.2 |
| 10. I needed to learn a lot of things before I could get going with this system. | 3.8 |
